# Diagnostic Accuracy of Ultrasonography-Guided Fine Needle Aspiration Cytology in Abdominopelvic Masses: A Prospective Study

**DOI:** 10.7759/cureus.41228

**Published:** 2023-06-30

**Authors:** Pratima Baisakh, Manas R Baisakh, B Shanta Kumari, Biswa B Mohanty, Dhiren K Panda, Swagatika Pradhan

**Affiliations:** 1 Anatomy, Institute of Medical Sciences and SUM Hospital, Siksha 'O' Anusandhan Deemed to be University, Bhubaneswar, IND; 2 Pathology, Apollo Hospitals, Bhubaneswar, IND

**Keywords:** abdominopelvic mass, ultrasonography, image-guided fnac, fine needle aspiration cytology, abdominal mass histologic correlation, histologic correlation, abdominal mass, histology, cytodiagnosis

## Abstract

Introduction

Accurate diagnosis of deep-seated abdominopelvic masses is crucial to distinguish malignant from non-malignant lesions for proper treatment and prognosis. Ultrasonography-guided fine needle aspiration cytology (USG-FNAC) is a cost-effective and straightforward procedure that offers rapid diagnosis, facilitating early initiation of treatment. This study aimed to determine the diagnostic accuracy of USG-FNAC in comparison to the biopsy diagnosis of various abdominopelvic masses in a resource-limited setting.

Materials and methods

This prospective study enrolled 208 patients with clinically and ultrasonographically confirmed abdominopelvic masses over two years. Of these, 64 cases were excluded from the study because of the non-availability of biopsy specimens. The remaining 144 cases comprised 88 males and 56 females, with a male-to-female ratio of 1.57:1. Patients’ ages ranged from 1.5 to 65 years, with most male patients aged 51 to 60 years and female patients aged 41 to 50 years. USG-FNAC was performed on these patients using a 22G spinal needle and a 10cc disposable syringe, and no complications were reported during the procedure. The cytological findings were compared to histopathological results when available. Dry smears were stained with May-Grunwald-Giemsa stain, while fixed smears were stained with Papanicolaou stain for cytological investigation.

Results

A total of 144 cases had both cytological and histological specimens available for comparison. The overall diagnostic accuracy of USG-FNAC was 90.97%, with 91.8% sensitivity for malignant lesions, 83.33% for benign lesions, and 85.7% for inflammatory lesions.

Conclusions

USG-FNAC provides high diagnostic accuracy for abdominopelvic masses, making it a valuable diagnostic tool in resource-limited settings. The technique allows for rapid diagnosis, triaging specimens for ancillary immunohistochemical and molecular studies, and in many cases, obviates the need for more expensive and time-consuming procedures like laparotomy and open biopsy.

## Introduction

Accurate diagnosis of deep-seated abdominopelvic masses is challenging, particularly in distinguishing malignant lesions from non-malignant ones, such as inflammatory lesions, due to their therapeutic and prognostic implications [1]. Various imaging techniques with increasing sensitivity can now easily identify even small abdominal lesions, which can be approached using image-guided fine needle aspiration (FNA) for precise diagnosis [1,2]. Two methods that commonly diagnose abdominal mass lesions are image-guided FNA and image-guided biopsy. FNA specimens are collected with 20- to 25-gauge needles for cytological examination. Although image-guided biopsies are generally preferred, FNA is popular due to its procedural simplicity and cost-effectiveness, particularly for deep-seated lesions, conditions where the needle must pass through the bowel, or when the lesion is close to a vessel [2-4]. FNA samples can be rapidly stained, enabling immediate assessment and diagnosis. Appropriate samples for other investigations, such as molecular and microbiology studies, can be triaged during the FNA procedure. This approach greatly impacts the avoidance of unnecessary surgical procedures and accelerates treatment planning. Ultrasonography (USG) is advantageous because it is relatively inexpensive, portable, and free of radiation hazards. It can be used to guide the needle in multiple planes. USG-guided FNA is a crucial diagnostic tool for solid or cystic and benign or malignant abdominopelvic masses. Percutaneous biopsy under imaging guidance is a popular and rapidly expanding interventional radiographic technique in imaging practice. Close cooperation between imaging and cytopathology departments ensures the most accurate diagnostic yield. Different studies report variations in the sensitivity of USG-guided FNA cytology (USG-FNAC) and biopsies [5-6]. The present study aimed to determine the diagnostic accuracy of USG-FNAC compared to the histologic diagnosis arrived through biopsy of various abdominopelvic masses.

## Materials and methods

This prospective study included patients with clinically and ultrasonographically confirmed abdominopelvic masses over two years. Institutional ethical committee approval was obtained from the Ethical Committee of the Institute of Medical Sciences and SUM Hospital (Approval No.: 412/21/02/2019). A total of 208 USG-guided FNA procedures were performed and evaluated for intra-abdominal masses. Exclusion criteria were patients with bleeding disorders, hepatic surface hemangioma, and echinococcosis. Patients with palpable abdominal lumps, radiologically appreciable non-palpable deep-seated small lesions, or failed blind FNA procedures were considered for inclusion. Patients’ demographics were recorded. USG-guided percutaneous fine needle aspiration cytology (FNAC) was performed using a 22G spinal needle and a 10cc disposable syringe. Smears were prepared from aspirated material; some were air-dried, while others were fixed with 95% ethyl alcohol. Air-dried smears were stained with May-Grunwald-Giemsa stain, and fixed smears were stained with Papanicolaou stain and hematoxylin and eosin (H&E) stain. In most cases, the surgical biopsy was performed later, and histopathology results were correlated with cytological findings.

## Results

The study included 208 FNA cases of intra-abdominal masses, with 64 cases (30.76%) excluded due to the unavailability of histological specimens for correlation. The remaining 144 cases comprised 88 males and 56 females, with a male-to-female ratio of 1.57:1 (Table 1). Patients’ ages ranged from 1.5 to 65 years, with most male patients aged 51 to 60 years and female patients aged 41 to 50 years (Table 1). Of the total 144 cases, 49 (34%) aspirations were from the right hypochondrium, followed by 37 (25%) cases from the right iliac fossa (Table 2).

**Table 1:** Age and gender distribution

Age group (years)	No. of cases	Male	Female
0-10	4	2 (1.38%)	2 (1.38%)
11-20	14	6 (4.16%)	8 (5.55%)
21-30	16	8 (5.55%)	8 (5.55%)
31-40	22	16 (11.11%)	6 (4.16%)
41-50	28	16 (11.11%)	12 (8.33%)
51-60	42	32 (22.22%)	10 (6.94%)
61 and above	18	8 (6.94%)	10 (5.55%)
Total	144	88 (62.51%)	56 (37.49%)

**Table 2:** Sites of aspiration by abdominal quadrant

Sites	Cases (n)	Percentage
Right hypochondrium	49	34%
Right iliac fossa	37	25%
Epigastrium	20	14%
Retroperitoneum	10	7%
Left iliac fossa	10	7%
Right lumbar	8	6%
Umbilical	6	4%
Left lumbar	4	3%

**Figure 1:**
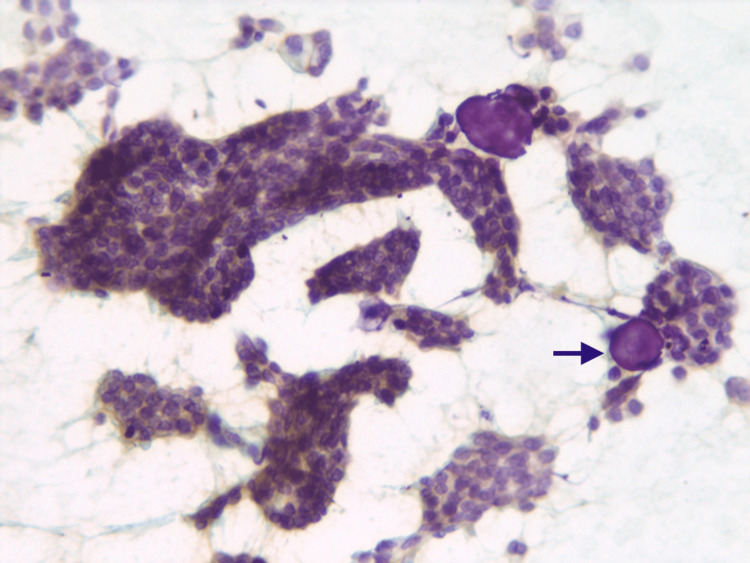
Papillary serous adenocarcinoma ovary Cytology smear shows tumor cells arranged in papillary clusters along with multiple psammomatous calcifications (black arrow) (200 x Pap). Abbreviation: Pap, Papanicolaou stain.

Cytological analysis of abdominal masses identified non-neoplastic/inflammatory lesions in 14 (9.72%) cases, benign lesions in six (4.16%) cases, and malignant lesions in 124 (83.33%) cases. Among non-neoplastic lesions, 10 were diagnosed as acute inflammatory lesions and four as tubercular lesions on cytology. Two suspicious cases were histologically diagnosed as gallbladder carcinoma (Table 3). Of the diagnosed benign lesions, six cases were identified as serous cystadenoma of the ovary; five were concordant with histology, while one was histologically identified as serous cystadenocarcinoma (Figure 1 and Table 4).

**Table 3:** Cytologic-histologic correlation of non-neoplastic lesions

Non-neoplastic lesion	Cytopathologic diagnosis (n)	Concordant histology (n)	Discordant histology (n)	Diagnostic accuracy
Acute pyogenic lesion	10	8	2	80%
Tubercular lesion	4	4	0	100%
Total	14	12	2	85.71%

**Table 4:** Cytologic-histologic correlation of neoplastic lesions

Malignancy status	Site	Lesion	Cytopathologic diagnosis	Concordant histology	Discordant histology	Diagnostic accuracy
Malignant	Liver	Hepatocellular carcinoma	28	26	2	92.85%
		Metastatic lesions	6	6	0	100
	Ovary	Mucinous cyst adenocarcinoma	6	5	1	83.33%
		Serous cyst adenocarcinoma	18	16	2	88.88%
	Stomach	Adenocarcinoma	10	10	0	100%
		Non-Hodgkin’s lymphoma	2	2	0	100%
	Colon	Adenocarcinoma	12	11	1	91.66%
		Germ cell tumor	12	10	2	83.33%
	Abdominal lymph node	Metastatic	4	4	0	100%
		Non-Hodgkin’s lymphoma	4	3	1	75%
	Gall bladder	Adenocarcinoma	12	11	1	91.66%
	Kidney	Renal cell carcinoma	5	5	0	100%
		Wilms’ tumor	2	2	0	100%
	Retroperitoneum	Soft tissue tumor	3	3	0	100%
	Total		124	114	10	91.93%
Benign	Ovary	Serous cyst adenoma	6	5	1	83.33%

**Figure 2:**
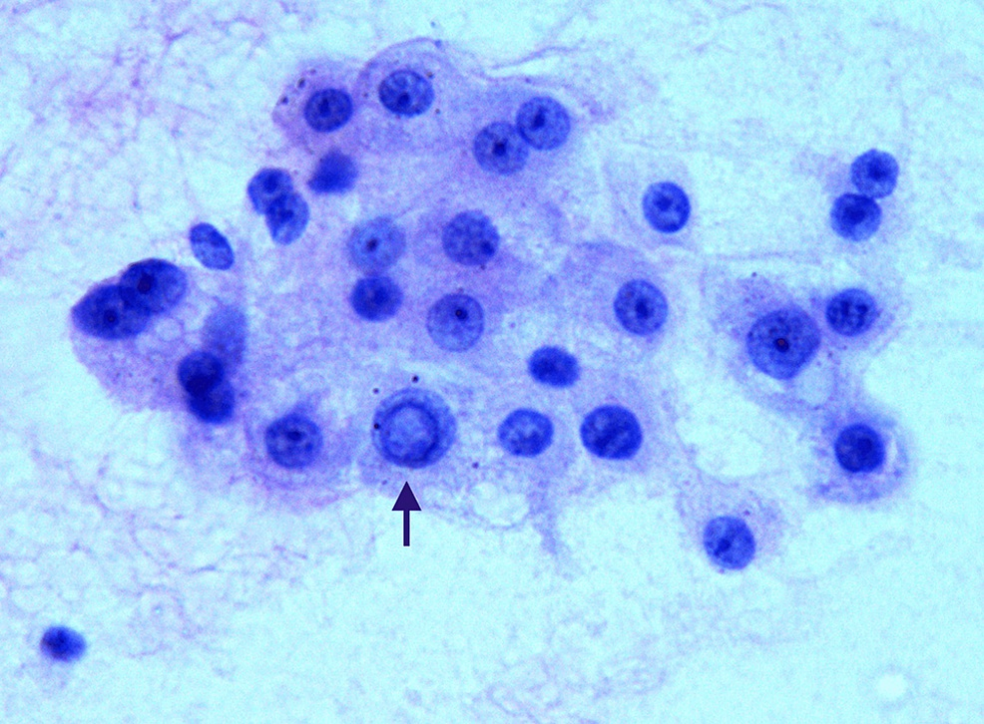
Well-differentiated hepatocellular carcinoma Cytology smear showing polygonal cells with a marked degree of anisokaryosis with nuclear vacuolation (black arrow), prominent nucleoli, and cytoplasmic bile pigments (400 x Pap). Abbreviation: Pap, Papanicolaou stain.

**Figure 3:**
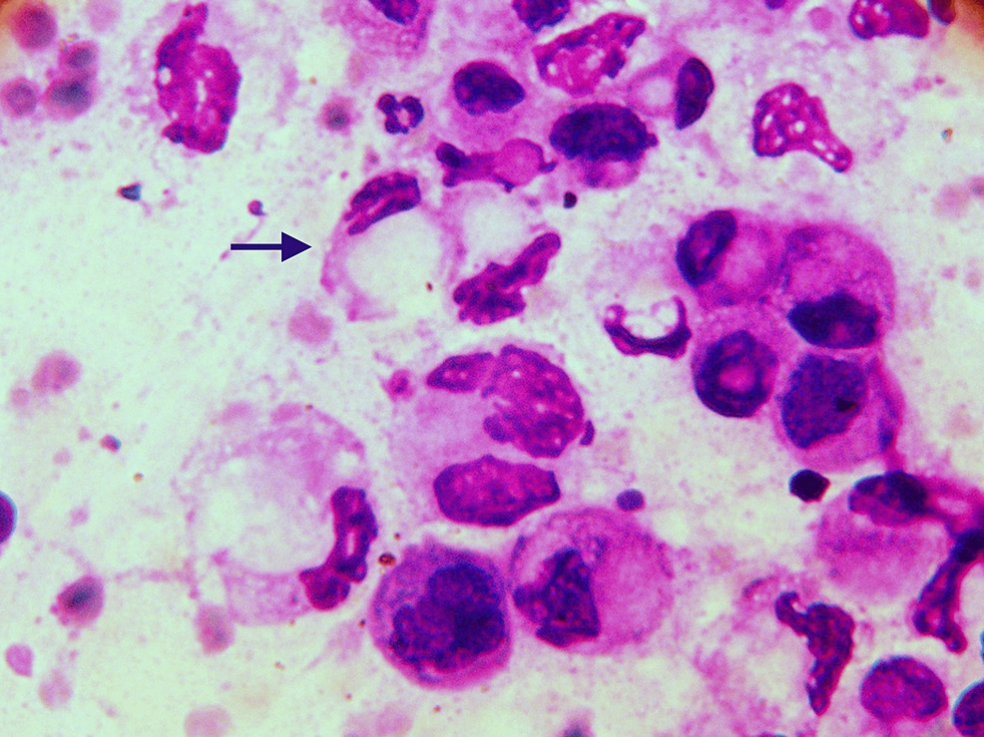
Adenocarcinoma stomach Cytology smear showing signet ring cell (black arrow) and marked cellular pleomorphism (400 x MGG). Abbreviation: MGG, May-Grunwald-Giemsa stain.

**Figure 4:**
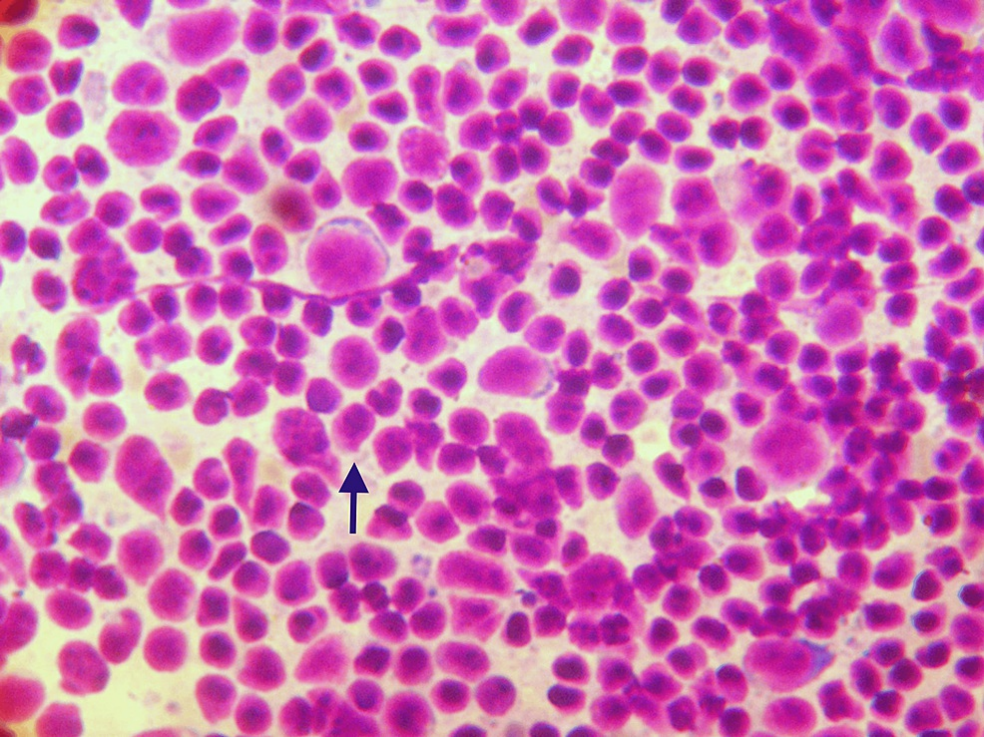
Non-Hodgkin’s lymphoma stomach Cytology smear reveals well-dispersed monomorphic cells (black arrow) (200 x MGG). Abbreviation: MGG, May-Grunwald-Giemsa stain.

**Figure 5:**
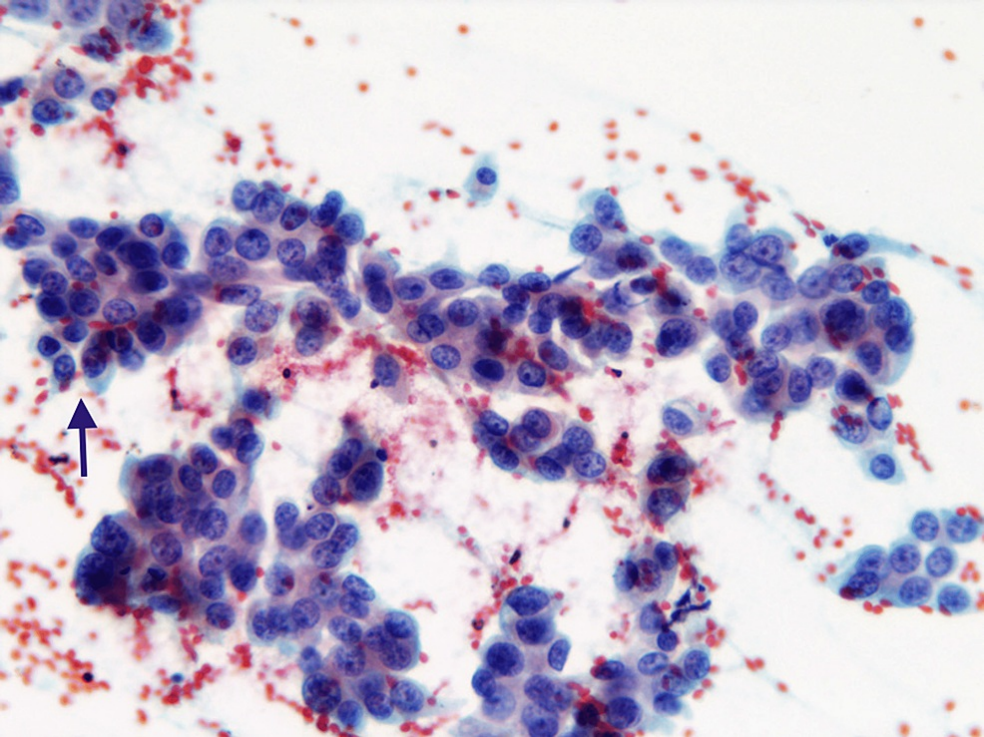
Renal cell carcinoma Cytology smear shows cells in cohesive sheets and papillary pattern with moderate pale cytoplasm (black arrow) and variable anisokaryosis (200 x Pap). Abbreviation: Pap, Papanicolaou stain.

Hepatocellular carcinoma was the most common malignancy among the 124 histologically confirmed malignant cases (Figure 2). Out of the 28 cases of hepatocellular carcinoma, 26 cases were consistent with cytodiagnosis. All six diagnosed metastatic liver lesions coincided with histological diagnoses. Other malignant lesions include adenocarcinoma and non-Hodgkin’s lymphoma of the stomach (Figures 3, 4), renal cell carcinoma (Figure 5), Wilms’ tumor, retroperitoneal soft tissue tumor, and secondary lymph node lesions, 100% consistent on histological evaluation.

**Figure 6:**
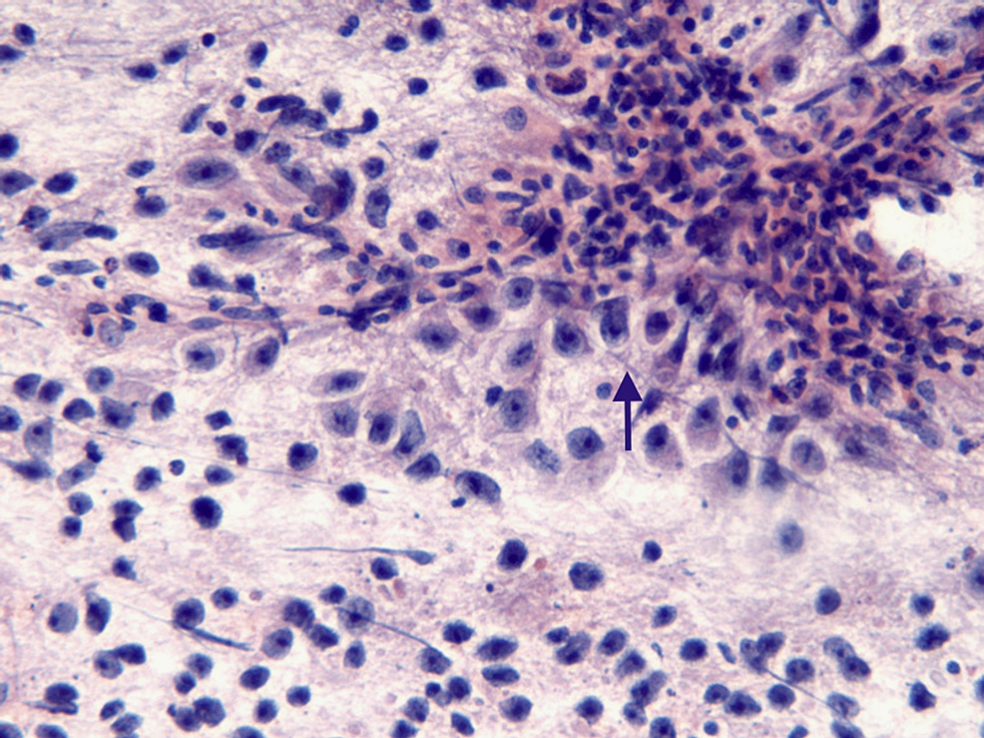
Dysgerminoma The microphotograph of the cytological smear shows dispersed polygonal cells with vesicular nuclei and prominent nucleoli (black arrow). Stromal fragments infiltrated with plenty of lymphocytes (200 x Pap). Abbreviation: Pap, Papanicolaou stain.

Of the 12 histologically proven gallbladder carcinoma, 11 cases (91.66%) were consistent with cytological diagnoses; the remaining cases had been diagnosed as acute cholecystitis. For adenocarcinoma of the colon, the diagnostic accuracy was 91.66%. Among the 12 germ cell tumor/dysgerminoma cases (Figure 6), 10 were consistent with histological diagnoses, while two were diagnosed as mucinous adenocarcinoma of the ovary. For serous cystadenocarcinoma, 16 cases (88.88%) corroborated with histological diagnoses, and the remaining cases were diagnosed as their benign counterparts (Table 4). The diagnostic accuracy was 91.79% for malignant lesions, 85.71% for inflammatory lesions, and 83.33% for benign lesions, with an overall diagnostic accuracy of 90.97%.

## Discussion

Intra-abdominal masses are commonly encountered lesions in clinical practice. Due to the wide variety of tissues within the abdominal cavity, diagnosing these lesions without radiological procedures and histopathological diagnoses is challenging. FNAC offers rapid diagnoses, helping to initiate early medical or surgical treatment and avoid invasive diagnostic procedures [7]. In the current study, USG was the imaging modality of choice for guided FNAC. However, computed tomography (CT) scan-guided aspirations can be performed for pancreatic, renal, and deep-seated retroperitoneal masses. Pancreatic and renal lesions are best aspirated under guidance through a posterior approach.

The age of the study population ranged from 1.5 to 65 years, in contrast to the study by Namshiker et al., where ages varied from 19 days to 89 years [8]. Another study by Glaxon et al. reported an age range from 14 to 90 years [9]. Most males were in the 51-60 years age group and most females were in the 41-50 years age group. The male-to-female ratio in the present study was 1.57:1, similar to studies by Namshiker et al. and Govind et al. [8-10]. The liver was the most frequently aspirated organ, followed by the ovary. This finding is similar to studies by Sheikh et al., Adhikari et al., and Nóbrega et al. [11-13].

Hepatocellular carcinoma was a more common malignancy than metastatic liver lesions. The sensitivity for hepatocellular carcinoma was 92.8%, and for metastatic lesions, it was 100% after comparison with biopsy findings. No complications were encountered during the aspiration procedure. Therefore, USG-FNAC can be carried out for hepatic lesions to reach a diagnosis where open biopsy is difficult, and the cost is an important factor. The next most common site for lesion aspiration was the ovary. Serous cystadenocarcinoma showed an 88.89% sensitivity, and mucinous cystadenocarcinoma showed an 83.33% sensitivity after histological confirmation. The most common lesion detected in the colon was adenocarcinoma, which reported a 91.66% sensitivity. Retroperitoneal soft tissue tumors and metastatic abdominal lymph nodes cytology showed a 100% sensitivity with histological findings. However, Gupta et al. argued that FNAC was unsuitable for diagnosing retroperitoneal soft tissue lesions [14]. Cytodiagnosis of non-Hodgkin’s lymphoma of both the stomach and abdominal lymph nodes aids in the early initiation of treatment as a life-saving measure.

The present study broadly categorized the entities into malignant (83.33% of total cases) and benign and inflammatory lesions (16.67%). This is higher than the findings of Smith et al. and Hemalatha et al., who reported 66% and 63.5%, respectively [15,16]. In benign lesions, all six cases were from the ovary. The present study reported an accuracy rate of 91.93% for malignant lesions, which is comparable to studies by Bobhate et al., Nautiyal et al., and Reyaz et al. [17-19] and is higher than previous studies [11,20,21]. There was an 85.71% accuracy for inflammatory lesions, which responded well to antibacterial and anti-tubercular drugs. The inflammatory lesions included liver abscess, tubo-ovarian abscess, appendicular abscess, lymphadenitis, and hydatid cyst of the liver. The sensitivity of the present study coincides with the study by Ahmad et al., who reported a 94.11% sensitivity [22]. The sensitivity of the present study was 91.8% for malignant lesions, 83.33% for benign lesions, and 85.7% for inflammatory lesions. The overall diagnostic accuracy was 90.97%. The present study revealed high accuracy and sensitivity of guided cytological diagnosis after comparing it with histological diagnoses for abdominopelvic lesions.

Our study had several important limitations. The sample size was relatively small, which limits the statistical power to detect significant differences and correlations among variables. We excluded 64 cases (30.76%) due to the unavailability of histological specimens for correlation, which could have introduced selection bias and affected the overall results. Also, our study focused solely on USG-FNAC, and it did not compare the diagnostic accuracy with other imaging modalities, such as CT scan-guided aspirations, potentially limiting the generalizability of the findings. Finally, our study was a single-center study, limiting the generalizability of the results to other settings and populations. Despite these limitations, our study provides insights into the diagnostic accuracy of USG-FNAC for abdominopelvic masses. Future research should address these limitations and explore the diagnostic accuracy of different imaging modalities in a larger and more diverse patient population.

## Conclusions

USG-FNAC provides high diagnostic accuracy for abdominopelvic masses, making it a valuable diagnostic tool in resource-limited settings. In low-income countries like India, where the cost-effectiveness of a procedure is crucial and evidence-based treatment is of prime importance, USG-FNAC provides optimal patient benefit. Its efficiency in arriving at a correct diagnosis, triaging specimens for cytology cell block for ancillary immunohistochemical and molecular studies (if required), and obviating the need for expensive and time-consuming procedures like laparotomy and open biopsy in many instances illustrate its diagnostic value.
